# Complete genome sequence of Indonesian probiotic strain *Lactiplantibacillus plantarum* subsp. *plantarum* Dad-13

**DOI:** 10.1128/mra.00118-24

**Published:** 2024-06-12

**Authors:** Endang Sutriswati Rahayu, Dian Anggraini Suroto, Mariyatun Mariyatun, Putrika Citta Pramesi

**Affiliations:** 1 University Center of Excellence for Integrated Probiotic Research and Application for Industry, Universitas Gadjah Mada, Yogyakarta, Indonesia; 2 Center for Food and Nutrition Studies, Universitas Gadjah Mada, Yogyakarta, Indonesia; 3 Faculty of Agricultural Technology, Universitas Gadjah Mada, Yogyakarta, Indonesia; The University of Arizona, Tucson, Arizona, USA

**Keywords:** buffalo milk, *dadih*, probiotic characteristics

## Abstract

Previous studies have investigated the probiotic properties of *L. plantarum* subsp. *plantarum* Dad-13. Nevertheless, genomic sequence data from previous studies were not yet available to support each probiotic characteristic. This study focused on the complete genome sequence of the strain to validate its role in specific probiotic properties.

## ANNOUNCEMENT

The *Lactiplantibacillus plantarum* subsp. *plantarum* Dad-13 (FNCC 0461) is a probiotic strain discovered by isolating it from *dadih*, a fermented milk product made from buffalo milk that originated in Minangkabau, Indonesia, in 1997. After 16s rRNA sequencing, the strain was identified as *Lactobacillus plantarum* (1), and its taxological name was changed to *Lactiplantibacillus plantarum* subsp. *plantarum*. Preceding studies have reported the survival ability in the gastrointestinal tract (2–4), safety (5), and physiological benefits (6–9) of the strain. Clinical studies have reported positive health effects of *L. plantarum* Dad-13 (10–12). The strain has been used in numerous probiotic foods such as fermented milk, yogurt, and chocolate. Although previous genomic analysis have been conducted (6, 7), the genome sequence analysis in this study further analyzes the strain using Nanopore to assemble a complete genome.

The sequencing process used default parameters for all software unless otherwise specified. The study used the first passage from the freeze-dried collection of the strain (FNCC0461) which was obtained from the Food and Nutrition Culture Collection. The strain was inoculated in de Man Rogosa Sharpe (MRS) broth media (Merck, Darmstadt, Germany; catalog number: 110661) and incubated at 37°C for 24 h. Then, one single colony was taken for sequencing.

The DNA of the strain was isolated using Zymo Research Quick-DNA HMW MagBead Kit (D6060 version) with ligation of sequencing adapters. DNA concentration was determined using both NanoDrop spectrophotometers and Qubit fluorometers. Library preparations, including DNA shearing, used Oxford Nanopore Technology kits. GridION sequencing was operated by MinKNOW software version 20.06.9. Base calling was performed using Guppy version 4.0.11 with high accuracy mode (13). All FASTQ files were filtered using Flitlong software (Ryan R. Wick, https://github.com/rrwick/Filtlong), and the quality was visualized using NanoPlot (14). *De novo* assembly and the circularization of contigs were conducted using Flye software version 2.8.1 (15). Then, the data were polished with Medaka software (ONT Research, https://github.com/nanoporetech/medaka). The assembled contig was aligned to the reference genome using Mauve version 2.4.0 (15) to detect any misassemblies or overlaps between contigs. Annotation was conducted using DFAST software (16) and RAST webserver (https://rast.nmpdr.org/). The assembled genomes and annotation were assessed using Busco (17) and CheckM (18) software. The annotated protein sequence was used for BLAST database (BLAST program from https://blast.ncbi.nlm.nih.gov/) to identify matches in the database for at least 90% identity and 90% length.

It must be noted that the genome assembly in this study was only performed using Nanopore reads where sequencing and assembly errors can occur. After the analysis, there was a total genome size of 3,404,719 bp, 44.3% G + C content, N50 value of 3,184,937 bp, 331 subsystems, 3,186 coding sequences, 2 contigs (with protein-encoding genes), and RNAs. Specific probiotic characteristic genes, according to FAO/WHO (19), are shown in Table 1.

**TABLE 1:** List of putative genes present in *L. plantarum* Dad-13 that are responsible for probiotic characteristics

Probiotic characteristic	Responsible genes in *L. plantarum* Dad-13	Protein identity (%)	Proposed function
Resistance to gastric acidity	dltA (WP_076631470.1), *Lactiplantibacillus plantarum*	100	D-alanine–poly(phosphoribitol) ligase subunit 1
	dltD (WP_003640704.1), *Lactiplantibacillus plantarum*	100	D-alanine transfer protein
	gadA, gadB, GAD (AHY84727.1), *Lactiplantibacillus plantarum*	100	Glutamate decarboxylase (gadB, gadA, GAD)
	clpL (WP_063488042.1), *Lactiplantibacillus plantarum*	100	ATP-dependent Clp protease ATP-binding subunit
Bile acid resistance	Cholylglycine hydrolase family protein (WP_063490672.1), *Lactiplantibacillus plantarum*	100	Choloylglycine hydrolase
	clpL (WP_063488042.1), *Lactiplantibacillus plantarum*	100	ATP-dependent Clp protease ATP-binding subunit
Adherence to mucus and/or human epithelial cells and cell lines	fbpA (PNW64149.1), *Lactobacillus* sp. ATCC 15578	100	Iron ABC transporter substrate-binding protein
	hslo (WP_003642088.1), *Lactiplantibacillus*	100	Molecular chaperone Hsp33
	htpX (WP_011101040.1), *Lactiplantibacillus plantarum*	100	Heat shock protein HtpX
	dnaK (WP_211757587.1), *Lactiplantibacillus plantarum*	97	Molecular chaperone DnaK
	puC (WP_128703834.1), *Lactiplantibacillus plantarum*	100	ABC transporter permease
	atpC (WP_016511441.1), *Lactiplantibacillus plantarum*	100	ATP synthase epsilon chain
	Elongation factor TU (WP_021732063.1), *Lactiplantibacillus*	100	Elongation factor Tu (tuf, TUFM)
Ability to reduce pathogen adhesion to surfaces (i.e., by competing with pathogens for surface adhesion)	fbpA (PNW64149.1), *Lactobacillus* sp. ATCC 15578	100	Iron ABC transporter substrate-binding protein
	hslo (WP_003642088.1), *Lactiplantibacillus*	100	Molecular chaperone Hsp33
	htpX (WP_011101040.1), *Lactiplantibacillus plantarum*	100	Heat shock protein HtpX
	dnaK (WP_211757587.1), *Lactiplantibacillus plantarum*	97	Molecular chaperone DnaK
	Elongation factor TU (WP_021732063.1), *Lactiplantibacillus*	100	Elongation factor Tu (tuf, TUFM)
	luxS (WP_003641031.1), *Lactiplantibacillus*	100	S-ribosylhomocysteine lyase
	cas4 (WP_003641444.1), *Lactiplantibacillus*	100	CRISPR-associated exonuclease Cas4
Bile salt hydrolase activity	Cholylglycine hydrolase family protein (WP_063490672.1), *Lactiplantibacillus plantarum*	100	Choloylglycine hydrolase
Assessment of metabolic activities—D-lactate production	ldhA (WP_003640741.1), *Lactiplantibacillus*	100	D-2-hydroxyacid dehydrogenase
Assessment of metabolic activities—bile salt deconjugation	Cholylglycine hydrolase family protein (WP_063490672.1), *Lactiplantibacillus plantarum*	100	Choloylglycine hydrolase

*L. plantarum* Dad-13 carried genes for acid tolerance, gamma-aminobutyric acid (GABA) production, stress tolerance, immunomodulation, and anti-inflammatory activity. Furthermore, it contains genes for bile resistance, D-lactate production, and DNA/protein protection. It harbors cas4 for virulence attack survival and a RiPP gene cluster related to bacteriocins (20). Stress-related protein genes enhance adhesion and high-temperature survival, while atpC and EF-Tu genes support cell adhesion properties. The strain’s digestive tract survival is aided by genes such as pbp2a, AraC transcriptional regulator, Na+:H+ antiporter, and nhaC. Its genome also contains genes involved in antidiabetic regulation, bile salt hydrolysis, and nutrient adaptation.

## Data Availability

The data sequence has been deposited and published at NCBI GenBank under the accession number JALLKE000000000 on 18 April 2022. The associated BioProject, BioSample, and Sequence Read Archive accession numbers are PRJNA832026, SAMN27771036, and SRR19627660, respectively.
